# Michigan State Board of Health

**Published:** 1882-02

**Authors:** 


### ARTICLE X.

MICHIGAN STATE BOARD OF HEALTH. Reported for the CHICAGO MEDICAL JOURNAL AND EXAMINER.

The regular quarterly meeting of this Board was held January 10, 1882, at its office in Lansing; the full Board being present. The Secretary presented his quarterly report, showing some of the work in the office during the past quarter. The quarter has been a very busy one, made so, in part, by the numerous outbreaks of diphtheria, scarlet fever and small-pox in the State, which has required much correspondence and the sending out of many documents. The compilation and issuing of the weekly bulletin of health in the State is now so systematized as not to take as much time as at first. It is published in probably two hundred newspapers in Michigan. In response to a request, fifty-seven health officers of villages have begun to make weekly roports of diseases. The Board reaffirmed the demand for these reports from health officers of cities. To each place in the State where diphtheria, scarlet fever, or small-pox was reported present, a letter was written to the health authorities, giving full instructions and suggestions how to prevent the spread of the disease. Documents containing elaborate and particular directions have been sent for free distribution throughout the vicinity. Each officer was requested to make a special report on the epidemic under his care, and some of the reports show how by determined action a contagious disease may be stamped out. The number of communications written during the quarter was 1,459. The number of diphtheria documents distributed was 29,000; of scarlet fever documents, 5,000; of general rules for restriction of contagious diseases, 6,000; reprints of weekly bulletins, 7,000. As showing the necessity for inspection and disinfection of immigrants, their clothing, baggage, etc., and especially for a system of surveillance to their destinations, a statement was made by the Secretary, of the introduction of typhus fever in Benzie county by Norwegian immigrants. The disease made its appearance over sixty days after the arrival of the immigrants, and spread quite freely (not being reported at the time or treated as a contagious disease by the local authorities), causing many cases of illness, and at least three deaths. The importance of inspection of immigrants at Port Huron, and of keeping those believed to be liable to spread communicable diseases under surveillance until their destination was reached, and then placing them in the watchful care of the local board of health, was freely discussed. As this Board has no funds available for such a purpose, the subject was referred to the President, Secretary and Dr. Lyster to confer with the National Board of Health, and take such action as is possible.

A report by Hon. LeRoy Parker, relative to duties of health officers in verifying diagnoses of contagious diseases, was read and ordered printed in the Annual Report. Mr. Parker reported the following: In Gaines township, Genessee county, a child of Mr. B. died of what a doctor called malarial fever, and did not report the case to the board of health, though it seems probable that it was really diphtheria. A neighbor and wife, Mr. and Mrs. B., assisted in preparing the corpse for burial. About the same time a child of Mrs. S. died from "sore throat," not reported as "dangerous to the public health," and some of the children of Mr. B. attended the funeral. Soon after Mrs. B. was taken sick with diphtheria, and in turn thirteen out of fourteen members of the family had it, and seven out of ten children died. The board of health promptly isolated this household, but the attending physician's error in diagnosis, or failure to report the first case, was fatal to the hopes of that family. In this connection the Board adopted the following preamble and resolutions:

WHEREAS, It is often difficult to recognize mild cases of diphtheria, or to distinguish such cases from a simple pharyngitis or laryngitis; and,

WHEREAS, Such mild cases of diphtheria often communicate a dangerous and fatal form of diphtheria;

*Resolved*, That it is the duty of physicians and householders in reporting diseases dangerous to the public health, and of local health authorities in their efforts to restrict such diseases, in every case to give the public safety the benefit of the doubt.

*Resolved*, That suspected cases of dangerous diseases should be reported, and precautionary measures should be taken.

Drs. Kellogg and Avery were appointed a special committee to report on the present knowledge of diphtheria, and Dr. Lyster was appointed a special committee to report upon the present knowledge of typhoid fever.

Mr. Parker reported that persons guilty of removing contagious disease placards from their houses could be punished, under the law which made the house in which the contagious disease was, a hospital, if declared so by the board of health, and subject to their rules and regulations. All rules and regulations of a board must first be published, then penalties may be inflicted for any violations.

Dr. Avery, as special committee on the subject, made a report relative to the overflowed lands in Gratiot and Clinton counties, and presented a resolution from the Board of Supervisors of Gratiot county. In accordance with the report, the Board adopted a preamble and resolution, as follows:

WHEREAS, The Board of Supervisors of Gratiot county has passed resolutions asking this State Board of Health to investigate the subject of the sickness caused by the overflow of Maple River, because of the dam at Maple Rapids, and "to advise the removal of said dam as being detrimental to the health of the communities living in the vicinity of said river;" therefore,

*Resolved*, That the Board of Supervisors of Gratiot county be informed that this Board has already had an investigation made, and from the report of such investigation is convinced that the dam at Maple Rapids causes a nuisance, and advises that, in case the owner of said dam will not remove the same, and thus abate the nuisance caused by the overflowing of land along said river, a bill in equity should be filed against the owner of said dam to compel him to remove the same.

The Secretary was directed to correspond with persons in some city in the western part of the State, relative to holding a second Sanitary Convention this winter. One will be held at Ann Arbor, February 28 and March 1. These conventions are held in accordance with invitations received from citizens, and under arrangements made by a local committee acting with a committee of this Board.

Dr. Hazlewood reported on the inspection of summer resort hotels as regards danger from fire, and asked if the present law providing for such inspection was not sufficient. Dr. Baker thought the law should be amended so as to take the inspection duties away from the political officers and place them among the duties of local boards of health. The question was referred to Mr. Parker, committee on legislation in the interests of health.

The Secretary presented a report of work of local boards of health, showing much good work done during the past season in the restriction of contagious diseases. He read letters showing the action of local boards of health with contagious diseases; one from J. R. Thomas, M.D., health officer of Bay City, relative to diphtheria; one from W. G. Elliott, M.D., health officer of Pontiac, relative to scarlet fever; and one from Foster Pratt, M.D., health officer of Kalamazoo, relative to small-pox.

The Secretary also read a *résumé* of work of other State Boards of Health, and it showed that typhoid fever was very widely prevalent; that small-pox was very prevalent in the Northern and Northwestern States, and that intermittent fever was present in Connecticut, Massachusetts and Rhode Island.

The next regular quarterly meeting of the Board will be April

11, 1882. There will probably be a special meeting of the Board in connection with the Sanitary Convention at Ann Arbor, February 28 and March 1, 1882.

---

METEOROLOGY AND CROUPOUS PNEUMONIA. — During the last three years, Dr. August Seibert has been carefully studying the combined influence of the various elements of the weather in producing croupous pneumonia. His conclusions have been deduced from a study of six hundred cases, observed in children under fourteen at the German Dispensary, New York.

By examining the meteorological phenomena of the three days prior to the reception of each of the cases, it was found that in seventy-eight per cent. the barometer was more or less strongly falling; in more than fifty per cent. it had fallen below the mean; in eighty-four per cent. the temperature was below 50° F.; in twenty per cent. the daily range of thermometer was 20° F. or more; in only four per cent. was there a strong falling thermometer; in fifty per cent. the wind was northerly; in thirty-three and one-half per cent. northwest, and in sixty-seven per cent. its velocity was fifteen miles an hour or more.

On comparing the lines of mean humidity and minimum temperature in comparative charts, it was found that in the months when pneumonia was the most frequent, the temperature was low and the humidity high. The greater the space between the lines, the more pneumonia; the less the space, the less the pneumonia.

From the facts gathered it may be concluded that—

1. A strong fall in the barometer;
2. A low thermometer;
3. A great velocity of wind;
4. A high humidity; and
5. Northerly winds, especially northwest, seem to favor the production of this disease.—*Am. Jour. of the Med. Sciences.*

